# Pasireotide as first line medical therapy for selected patients with acromegaly

**DOI:** 10.1007/s11102-025-01514-3

**Published:** 2025-04-11

**Authors:** Nicoleta C. Olarescu, Anders P. Jørgensen, Shahriar Atai, Markus K.H. Wiedmann, Daniel Dahlberg, Jens Bollerslev, Ansgar Heck

**Affiliations:** 1 Section of Specialised Endocrinology, Clinic of Medicine, Oslo University Hospital, Oslo, Norway; 2 Faculty of Medicine, University of Oslo, Oslo, Norway; 3 Department of Neurosurgery, Oslo University Hospital, Oslo, Norway

**Keywords:** Somatostatin analogue, Somatostatin receptor ligand, Pituitary, Octreotide test

## Abstract

**Background and purpose:**

In acromegaly, growth hormone (GH) excess and pituitary tumours are typically managed through transsphenoidal surgery, often in combination with somatostatin receptor ligands (SRLs) given either before or following surgery. Although first-generation SRLs (lanreotide and octreotide) are efficacious in many patients, some exhibit resistance.

**Methods:**

We present the efficacy of the second-generation SRL, pasireotide, in six patients anticipated to be resistant to first-generation SRLs. The patients had large, hyperintense tumors on T2-weighted MRI and sparse granulation pattern by histology.

**Results:**

Over three to eight months, pasireotide reduced tumour volume in all patients and improved GH and IGF-1 levels. Visual field defects normalised. Despite hyperglycemia, requiring antidiabetic treatment in two patients, pasireotide proved effective as a first pharmacological therapy.

**Conclusion:**

This series supports the use of pasireotide for rapid tumour control and GH reduction, in selected patients with complex and large tumours, likely to be resistant to first-generation SRLs. This approach expands the therapeutic options for managing the most challenging cases enhancing the potential for other subsequent treatment modalities.

## Introduction

Most patients with acromegaly are managed effectively by transsphenoidal surgery, often combined with pharmacological therapies targeting GH overproduction. A cornerstone of medical therapy is the use of somatostatin receptor ligands (SRL), which possess both anti-secretory and anti-tumor properties [1].

The first generation (fg) SRL lanreotide and octreotide achieve disease control in many patients [2, 3]. However, a subgroup of mostly younger patients with large and invasive tumors, remain particularly challenging to manage. These complex tumors are difficult to treat surgically with an increased risk of complications compared to non-invasive tumors [4].

Pasireotide is a second-generation (sg) SRL with the highest affinity for somatostatin receptor subtype 5 (SSTR5), followed by SSTR2. As by Summary of Product Characteristics (SPC), pasireotide is typically considered in patients resistant to fg SRL [5]. Histological characteristics and T2-weighted MRI can predict resistance to fg SRL [6, 7]. Previous studies have primarily assessed efficacy of pasireotide as a second- or third-line pharmacological treatment [5]. However, pasireotide may be a viable option as first-line therapy in patients anticipated to be resistant to fg SRLs. Preoperatively, the choice of fg or sg SRL can be based on hyperintensity T2-weighted MRI signal, and blunted response to an acute octreotide test [8]. If histological samples are available, sparse granulation pattern and SSTR status are predictive factors to SRL efficacy [8, 9].

Here, we present short-term outcomes of biochemical and tumor volume response in six highly selected patients treated with pasireotide, as their first pharmacological treatment for acromegaly.

## Materials and methods

### Study design and participants

The present report is an observational case series of highly selected patients from a tertiary pituitary care centre. All patients consented to inclusion to the pituitary registry at Oslo University Hospital and the study was approved by the regional ethical committee (REK 2009/1560 and 15240).

### Patients

Six patients with newly diagnosed acromegaly and evaluated to be resistant to fg SRLs were treated with pasireotide pamoate, as the first pharmacological therapy.

The selection criteria included the presence of large and invasive tumors with T2 hyperintensity on MRI, a blunted response to an octreotide test-dose, sparsely granulated somatotroph adenomas on immunohistochemistry (if available), or a combination of these (Table 1) [7, 8, 10]. Five patients had a tumor classified as Knosp-Steiner grade 4, one (#5) grade 2.

**Table 1:** Baseline characteristics, treatment duration and outcomes

		BaselinePasireotide			Follow-up							
ID_sex,ageatdiagnosis	Indication for pasireotide as first line medical treatment	MRI [ml]	IGF-1 /ULN	GH [µg/L]	MRI [ml]	Δ MRI [%]	IGF-1 /ULN	ΔIGF-1 [%]	GH [µg/L]	Δ GH [%]	Months until MRI	Months until lab.
#1_F, 31	Histology / T2 hyper / Oct.test	7,8	1,82	5,2	4,9	-38	0,89	-51	2,1	-57	6,2	8,4
#2_M, 51	T2 hyper	47,4	2,73	2,9	27,2	-43	2,64	-3	2,7	-90	6,6	6,6
#3_F, 35	T2 hyper / Oct.test	7,3	1,90	1,6	4,3	-41*	1,34	-29*	1,5	-65*	4,5	8,1
#4_F, 30	Histology / T2 hyper	9,4	3,93	59	4,5	-52	2,79	-29*	3,8	-15*	5,0	4,0
#5_F, 53	T2 hyper / Oct.test	10,0	2,23	17	5,2	-49	1,95	-12	3,3	-36	5,5	5,5
#6_F, 50	T2 hyper	25,8	4,31	22,5	16,8	-35	0,95	-78	0,9	-95	2,8	3,6

F: female; M: male; T2 hyper: Tumor hyperintensity on T2 weighted MRI; Oct.test: Octreotide test; lab.: laboratory assessment

* #3: add-on dopamine agonist (cabergoline) from month 2; #4: combined effect of TSS and pasireotide

In four patients, primary pituitary surgery was initially considered. However, based on the multidisciplinary team (MDT) assessment, it was concluded that primary transsphenoidal (TSS) or transcranial debulking surgery would carry significant risks. Consequently, a trial with pasireotide was initiated as first-line treatment to potentially reduce tumor volume and alleviate GH excess.

In the remaining two patients, pasireotide 20–40 mg every four weeks was given following transsphenoidal tumor debulking.

An acute octreotide test (50 µg s.c.) was performed in three patients revealing a GH decrease < 75% [11].

### Measurements and assessments

Outcome measures were the changes in tumor volume and biochemical markers (GH and IGF-1) after treatment with pasireotide.

Routine ophthalmologic exams including visual field assessment were performed in all patients before and after pasireotide treatment.

GH and IGF-1 are accredited analyses at the Department of Clinical Biochemistry, Oslo University Hospital (OUS) and were analysed as part of the clinical routine. The tumor volume was estimated by the formula width*height*length*0.5. The tumor invasiveness was assessed based on the Knosp-Steiner classification [12].

Histological samples were analysed by the clinical-pathological routine workup (Department of Pathology, OUS) with staining of pituitary transcription factors, GH, prolactin, Cytokeratin (CK) 8 and Ki67.

## Results

**Fig. 1:**
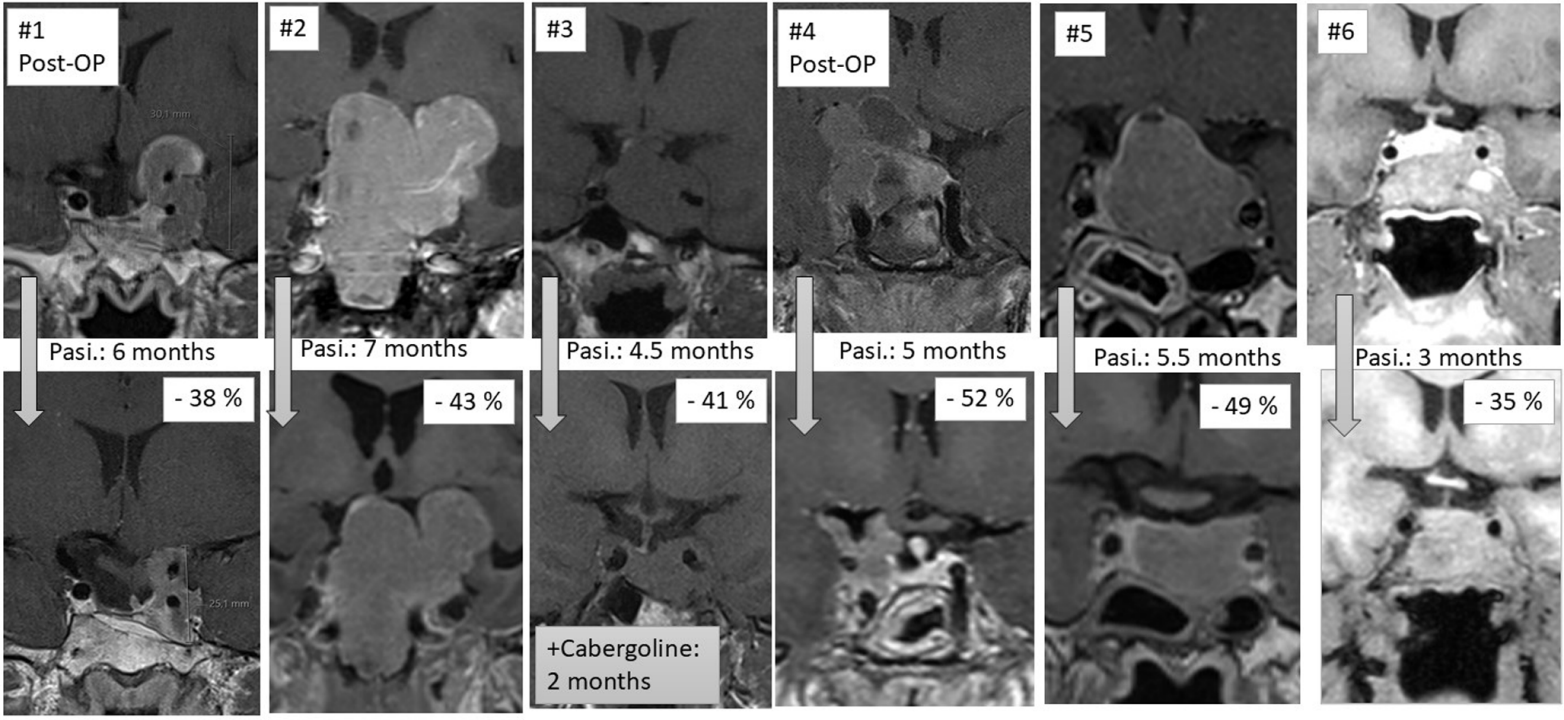
MRI before pasireotide treatment (upper row) and after 3–7 months. Tumor volume reduction as assessed by ellipsoid formula (lower row). MRI with T1-weighted, contrast enhanced series, except for #3 without contrast enhancement

Pasireotide as first line medical therapy for a period of three to eight months resulted in tumor volume reduction ranging from 35 to 52% (Table 1; Fig. 1). GH and IGF-1 levels improved in all patients with a reduction range of 15–95% (GH) and 3–78% (IGF-1). Tumor volume reduction was independent of biochemical improvement.

In four patients, visual field impairment was found prior to treatment. In all four patients, the visual field normalised after pasireotide treatment (#2, #6), or after TSS followed by pasireotide (#1, #4).

HbA1c was within reference range (20–42 mmol/mol) before treatment in all patients. One patient received antidiabetic medication before initiation of pasireotide. In this patient HbA1c increased from 34 to 52 mmol/mol. In another patient, HbA1c increased to the diabetic range (48 mmol/mol).

## Discussion

Pasireotide as a first pharmacological approach in this series of patients with newly diagnosed acromegaly and presumed resistance to fg SRL, led to tumor control, normalized visual field and improved biochemical activity in all patients within a few months. The combined effects in patients with large tumors not accessible for curative surgery, are an important early achievement in the multimodal management.

Patients with large, T2 hyperintense tumors and sparsely granulated somatotroph adenomas are the most challenging to manage in terms of controlling GH excess and tumor growth. Despite multimodal treatment, disease control remains difficult for this subgroup, and many cases are defined as aggressive pituitary tumors [3, 13].

In the early head-to-head clinical trial comparing fg SRL to pasireotide as primary medical treatment, no significant difference in tumor volume reduction was found [14], but pasireotide was given to an unselected group of patients. However, in a previously published case with similar characteristics as in our series, the treatment resulted in approximately 50% tumor volume reduction [15]. In another case, a treatment naïve patient with a GH producing microadenoma received pasireotide as the first treatment. This patient achieved biochemical remission and complete regression of the pituitary adenoma [16]. According to the SPC for pasireotide, the indication is limited to patients “for whom surgery is not an option or has not been curative and who are inadequately controlled on treatment with another somatostatin analogue” [17]. This indication is based on previous phase 3 studies on unselected patients with acromegaly [14], and those with proven treatment failure of fg SRL [18]. Clinical studies with the selection criteria as presented in our cases have so far not been conducted.

The potential of T2 weighted MRI and IHC to predict response to medical therapy with fg and sg SRL has previously been demonstrated [7, 19, 20]. We applied this knowledge to patients with a high surgical risk and presumed fg SRL resistance, as proposed previously [6]. SSTR2 immunoreactive score (IRS) has previously been proven to be predictive for the response to fg and sg SRL, but was unfortunately not available in the present study [8, 21, 22]. Further, we used blunted response to an octreotide test dose as indicator for fg SRL resistance, however, test procedures differ, and cut-off values are not universally validated and thus not generalizable [8].

We demonstrate the feasibility and safety of a tailored approach with pasireotide as the first medical treatment in highly selected patients. The present report focuses on the initial treatment effect, but all patients will require further multimodal treatment. We believe that further treatment will be facilitated after the initial tumor volume and GH reduction: the reduced tumor volume eases surgery and radiation therapy, and by a lower GH excess the likelihood of achieving biochemical control increases. An approach with fg SRL as first pharmacological treatment might have delayed effective therapy and would not have been justifiable.

Hyperglycemia is a concern with pasireotide treatment and led to initiation of antidiabetic treatment in two of the patients. Adequate monitoring and management of hyperglycemia is warranted and feasible [23]. We consider the positive treatment effects on tumor size and GH excess to outweigh this manageable side effect.

## Conclusion

In highly selected patients with acromegaly characterized by large tumors resistant to fg SRLs, pasireotide may be a first line medical treatment. This approach facilitates early tumor control, reduces GH excess, and may ease subsequent multimodal treatment.

## Data Availability

No datasets were generated or analysed during the current study.
